# Pulsed 980 nm short wavelength infrared neural stimulation in cochlea and laser parameter effects on auditory response characteristics

**DOI:** 10.1186/s12938-015-0084-7

**Published:** 2015-10-07

**Authors:** Jingxuan Wang, Jianren Lu, Chen Li, Lei Xu, Xiaofei Li, Lan Tian

**Affiliations:** School of Information Science and Engineering, Shandong University, 250100 Jinan, Shandong China; Shandong Artificial Auditory Engineering Centre, 250022 Jinan, Shandong China

**Keywords:** Short-wavelength infrared (SWIR), Optical neural stimulation, Cochlea, Optically evoked ABRs

## Abstract

**Background:**

Auditory neural stimulation with pulsed infrared radiation has been proposed as an alternative method to activate the auditory nerves in vivo. Infrared wavelengths from 1800–2150 nm with high water absorption were mainly selected in previous studies. However, few researchers have used the short-wavelength infrared (SWIR) for auditory nerve stimulation and limited pulse parameters variability has been investigated so far.

**Methods:**

In this paper, we pioneered to use the 980 nm SWIR laser with adjustable pulse parameter as a stimulus to act on the deafened guinea pigs’ cochlea in vivo. Pulsed laser light was guided through the cochlear round window to irradiate the spiral ganglion cells via a 105 μm optical fiber, and then the laser pulse parameters variability and its influence to auditory response characteristics were studied.

**Results:**

The results showed that the optically evoked auditory brainstem response (OABR) had a similar waveform to the acoustically induced ABR with click sound stimulus. And the evoked OABR amplitude had a positive correlation, while the OABR latency period showed a negative correlation, with the laser pulse energy increase. However, when holding the laser peak power constant, the pulse width variability ranged from 100 to 800 μs showed little influence on the evoked OABR amplitude and its latency period.

**Conclusions:**

Our study suggests that 980 nm SWIR laser is an effective stimulus for auditory neurons activation in vivo. The evoked OABR amplitude and latency are highly affected by the laser pulse energy, while not sensitive to the pulse width variability in 100–800 μs range.

## Background

Pulsed infrared neural stimulation (INS) has been investigated as an alternative to electrical stimulation to evoke neural activity [1–3]. The INS technique can activate neurons directly without the optogenetic modification or other additional interventions [4, 5]. And its features such as contact-free, no stimulus artifact and spatial resolution improvement may bring advantages over the electrical neural stimulation [6, 7]. INS method has also been used in auditory system stimulation which could bring beneficial to cochlear implants [8, 9]. Relative researches have demonstrated that INS with appropriate parameters is an effective tool to trigger hearing response. A 2120 nm Ho:YAG laser system was firstly applied to evoke the gerbil’s auditory nerves and the laser light was delivered by a 200 μm diameter optical fiber to irradiate the spiral ganglion cells through the round window of cochlea [10]. Another study on acoustic events induced by infrared laser was implemented with an 1850 nm pulsed laser on cochlea of normal hearing rats [11]. Littlefield used the single-fiber recording approach to characterize the single auditory nerve response to optical stimulation of the 1844–1873 nm laser wavelengths [12].

The mechanisms of the laser-tissue interaction have been the topic of discussion in relevant literatures. Although the optical neural stimulation mechanisms have not been fully explained, it is generally considered that the photo-thermal interaction due to water absorption played an important role in INS [6, 13]. In this process, the water in tissue absorbed the pulsed laser energy and converted into heat, which resulted in the heat gradient transient. And the heating transient of neurons may lead to the heat-sensitive ion channels (TRPV channels) activation or a change in ion channels conductance, which evoked the nerve response [14, 15]. Besides, it has been discussed that the cochlear response to laser radiation might be dominated by an acoustic event resulting from a pressure wave acting on hair cells when using the normal hearing animals [11, 16–18]. Wenzel et al. demonstrated the hair cell mediated response to green laser stimulation with normal hearing guinea pigs [19]. Therefore, it is essential to eliminate the hair cells function in INS studies [8, 10, 11], thus to ensure the direct cochlear neural response from optical stimulation. In our study, deafening procedure was implemented to exclude the pressure-wave-induce acoustic response and ensure the direct interaction between laser and neurons [20].

Although experimental purposes and subjects are various in the relevant optical auditory stimulation researches, the radiation wavelength was selected mainly between 1.8 to 2.2 μm, due to its high absorption coefficient of water [21, 22]. Nevertheless, researchers in Chongqing University proposed that 808 nm short-wavelength infrared (SWIR) laser with lower water absorption coefficient and deeper tissue penetration depth could pass through the perilymph fluid filled cochlea and activate the auditory nerves effectively [20]. The study verified the feasibility of the auditory neural stimulation with SWIR laser. Nevertheless, the pulse parameters variability and its effects on auditory perception characteristics with SWIR stimulation need for further study. In this study, our team pioneered to select the SWIR laser with the central wavelength of 980 nm as a stimulus to evoke the auditory nerves in cochlea. Guinea pigs were chosen as experimental animals and additional deafening procedures were performed before optical stimulation to eliminate the hair cells function. So that we could ensure that the SWIR radiation directly activated the auditory nerves. The pulsed SWIR laser light was coupled into a 105 μm diameter optical fiber for delivery. The distal tip of fiber was placed near the round window and oriented toward the modiolus to irradiate the spiral ganglion cells in the basal turn of cochlea. The influence of pulse parameters (pulse energy, pulse width) to the auditory response was further studied by recording and analyzing the intensity and latency period of optically evoked auditory brainstem response (OABR). This paper aims to investigate the effect of pulse parameter variability on the optical evoked auditory neural response characteristics, when stimulating the neurons with short-wavelength infrared laser.

## Methods

### Animal model and preparation

Guinea pigs are widely used in researches on auditory and inner ear disease due to the sensitive audition [16, 23, 24]. In this study all experiments were operated in vivo with adult guinea pigs of either sex. The whole animal experiment operations were conducted in accordance with the Guidance for Care and Use of Laboratory Animals of Shandong University and with support from the otorhinolaryngology department of Shandong Provincial Hospital.

**Fig. 1:**
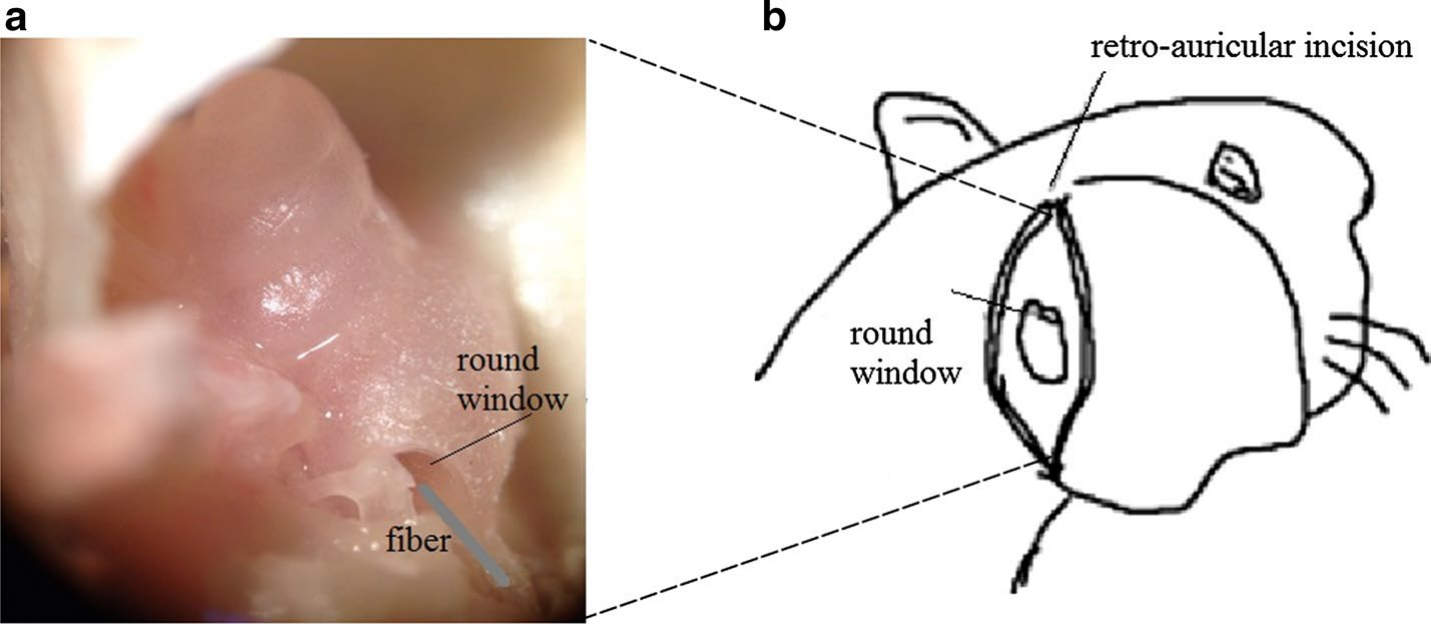
Diagram of the experimental surgery setup. **a** The placement and orientation of the optical fiber. The fiber was inserted into the round window and oriented to the modious. **b** The retro-auricular incision at the right side of the animal’s head

The animals were anesthetized by single intraperitoneal injection of ethyl carbamate solution (1 g/kg body weight in 20 % sterile saline) in first step. Additional dose of 0.2 ml could be needed to ensure deep anesthesia of guinea pigs during the experiment. Once the animals were under deep anesthesia condition, they were then positioned on an operation table with animal heads stabilized in a head holder, preparing for the otologic surgical procedure. Considering the stability of the experiment and animal care, animals were placed in the thermostatic chamber to maintain body temperature at 38 °C. Next, the surgery was carried out. The animal’s bulla was exposed by retro-auricular incisions to provide access to the cochlea. Then the muscle tissue and soft tissue were dissected and the bulla’s posterior-lateral part was opened to get access to the round window niche. Figure 1b shows the surgical incision location and the exposed round window of cochlea.

### ABR measurement

In this study the record of ABR was used as the index of auditory response. The Nicolet evoked potentials system (Endeavor CR, Nicolet Biomedical, USA) was used to record and measure the ABR signals. Three subdermal needle electrodes (DSN-1248 type, 13 mm length, 0.4 mm diameter, SunSpots series, Axon System, Inc. USA) were placed under the skin to obtain the ABRs. The record electrode was placed at the vertex of animal head, and the reference electrode was placed in the ipsilateral mastoid, while the ground electrode was placed in the neck muscles. Each acquired ABR data was filtered by the digital filter between 100 to 3000 Hz and averaged from 1024 trials in the Nicolet instrument to remove the irrelative noise and interference. All the recording operations were conducted in an electromagnetic shielding and sound-proof room.

### Acoustic stimulation

After the surgery, we firstly gave the animals acoustic stimuli, serving as a reference to the later SWIR laser stimulation. The whole experiment was performed in the sound-proof circumstance to keep the results reliable.

The inner trigger acoustic stimulator of the Nicolet Evoked Potentials system was used to stimulate the inner ear. The sound stimuli were delivered to the animal right ear canal with a polyurethane foam earplug, which was coupled to a transducer (TIP300 type, Endeavor CR, Nicolet Biomedical, USA) via a silicone tube. The transducer was attached to the acoustic stimulator of the potentials system and it was calibrated with a digital phonometer before each measurement. A series of 11 Hz click-sound stimuli with alternating polarity were used in the study, which was normally used in relevant studies [19, 20]. The acoustically induced ABR at several sound pressure level (SPL) from 40 to 100 dB SPL in 20-dB steps were recorded, respectively. During the acoustic stimulation, the vibration of periosteum was transmitted through the middle ear to the cochlea, and then perceived by the auditory neurons.

### Deafening procedure

Given that photoacoustic-induced vibration may also induce the auditory neural response in normal hearing animals [11, 16–18], laser stimulation with deafened animal is indispensable in order to verify the direct activation of auditory neurons by the SWIR laser. Therefore after the acoustic stimulation operations in normal hearing animals, extra deafening procedure was taken to deafen the guinea pigs’ cochlea acutely. The osseous spiral lamina was exposed carefully with a medical electrical drill and the basilar membrane was destroyed subsequently with a dissecting needle. The invasive procedure blocked the hair cells’ sensory function and caused hearing loss. Extra acoustic stimulation tests were done after the deafening operation to verify the loss of hearing. Then the laser stimulation was performed immediately after the acoustic test.

### Laser stimulation

The pulsed SWIR laser with the central wavelength of 980 nm was used for the optical neural stimulation in vivo. The laser light was coupled into an optical fiber (Flexi Ray series, 105-μm-diameter core, Art photonics, Germany) for delivery. The laser pulse parameters, including pulse energy and pulse width, can be regulated with the adjustable current source and the real-time data can be monitored through a human machine interface.

Here in our study, we chose the modiolus of the cochlea as the stimulation site. When the round window was exposed after the animal surgery, the optical fiber was carefully inserted into the round window membrane using a three-axis micromanipulator (TSD-40 XYZ, SIGMA KOKI, Japan). Fiber position was carefully adjusted to make it directly oriented to the modiolus, the distance from the fiber distal end to the stimulated spiral ganglion cells was approximately 0.3 mm. Figure 1a shows the orientation and placement of the optical fiber towards the round window of the cochlea. Optical fiber was cleaned and cleaved before each measurement. The modulated pulsed laser light was then transmitted by the optical fiber and guided into the basal turn of the cochlea to irradiate the spiral ganglion.

In the first step, the effect of SWIR laser pulse energy to its evoked auditory neural response was investigated. The laser parameters of repetition rate and pulse width were kept to 11 Hz and 200 μs. And the laser single pulse energy was ranged from 0.05 to 0.5 mJ by adjusting the laser output peak power from 0.25 to 2.5 W. The pulse energy was measured in air with an energy meter (Nova II meter, PE10BF-C probe, Ophir Photonics, Israel) at the tip of optical fiber. The OABRs evoked with different laser pulse energy levels were recorded accordingly and the ABR wave III peak amplitude and the wave III absolute latency time were measured for later analysis.

Furthermore, the SWIR laser pulse width variation and its effects on neural response were studied. The laser pulse repetition rate remained 11 Hz and the peak output power was kept constant at 1 W during the stimulation process. Then the pulse width was adjusted from 100 to 800 μs in 100-μs steps. The OABR wave III peak amplitude and the wave III absolute latency time at each laser pulse width were recorded and measured subsequently.

### Data statistical method

Each ABR data was averaged from 1024 trials and synchronized at the stimuli start point, for both acoustic and optical stimulations. In this study, the ABR waveforms were plotted with the peak positive voltage input of the amplifier with the recording electrode in the vertex site. And the wave III peak amplitude was used as the representation of auditory neural response intensity. Meanwhile, the time interval between the start point and the peak of wave III was plotted as wave III absolute latency time. The Origin 8.6 software was used for data processing and analysis. The ABR data through all experimental animals were averaged, with the mean, standard deviation and standard error of data calculated under the different stimulation parameters. The one-way analysis-of-variance (ANOVA) statistical model was implemented to verify if the value differences between individual animals were significant in the OABR parameters.

## Results

A total number of four animals were evaluated in the present study. The acoustically evoked ABR (AABR) was recorded from all four animals before the deafening procedure, and the click-sound stimuli were used with varying levels from 40 to 100 dB SPL. The ABR threshold was defined as the lowest stimulation level which could generate visually detectable waveforms beyond the background noise level.

**Fig. 2:**
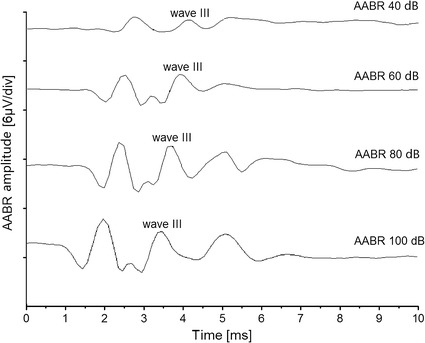
ABRs to increasing levels acoustic stimuli from 40 to 100 dB SPL with normal hearing animals. The AABR wave III peak was plotted in the traces

**Fig. 3:**
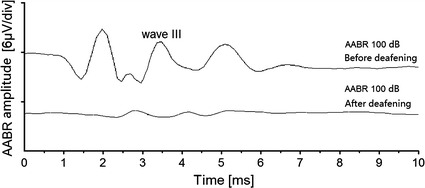
ABRs to 100 dB SPL acoustic stimuli before and after the deafening procedure. No detectable AABR waveforms could be recorded after the deafening surgery

Evoked AABRs under different acoustic stimulation levels were presented in Fig. 2. The AABRs all showed the classical Jewett waveform. Along with the SPL increasing, AABR intensity was strengthened, with the wave III peak amplitude from 0.64 μV at 40 dB SPL to 3.55 μV at 100 dB SPL. While after the deafening procedure, which destroyed the cochlear basilar membrane and hair cells, no detectable AABR waveform was recorded even at 100 dB stimuli. This indicated that the animals’ hearing function has already lost (shown in Fig. 3).

**Fig. 4:**
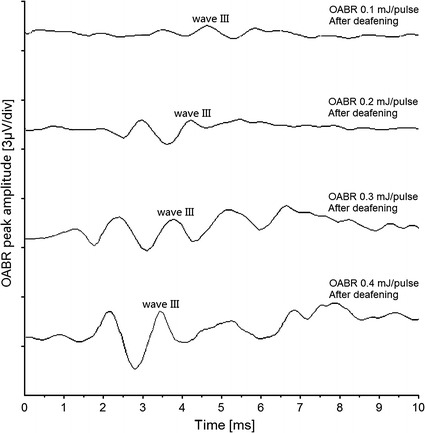
OABRs evoked by varied pulse energy levels of SWIR laser after the deafening procedure with deafened animal. The laser repetition rate was set 11 Hz and the pulse width was kept 200 μs. The OABR wave III peak was plotted in the traces

**Fig. 5:**
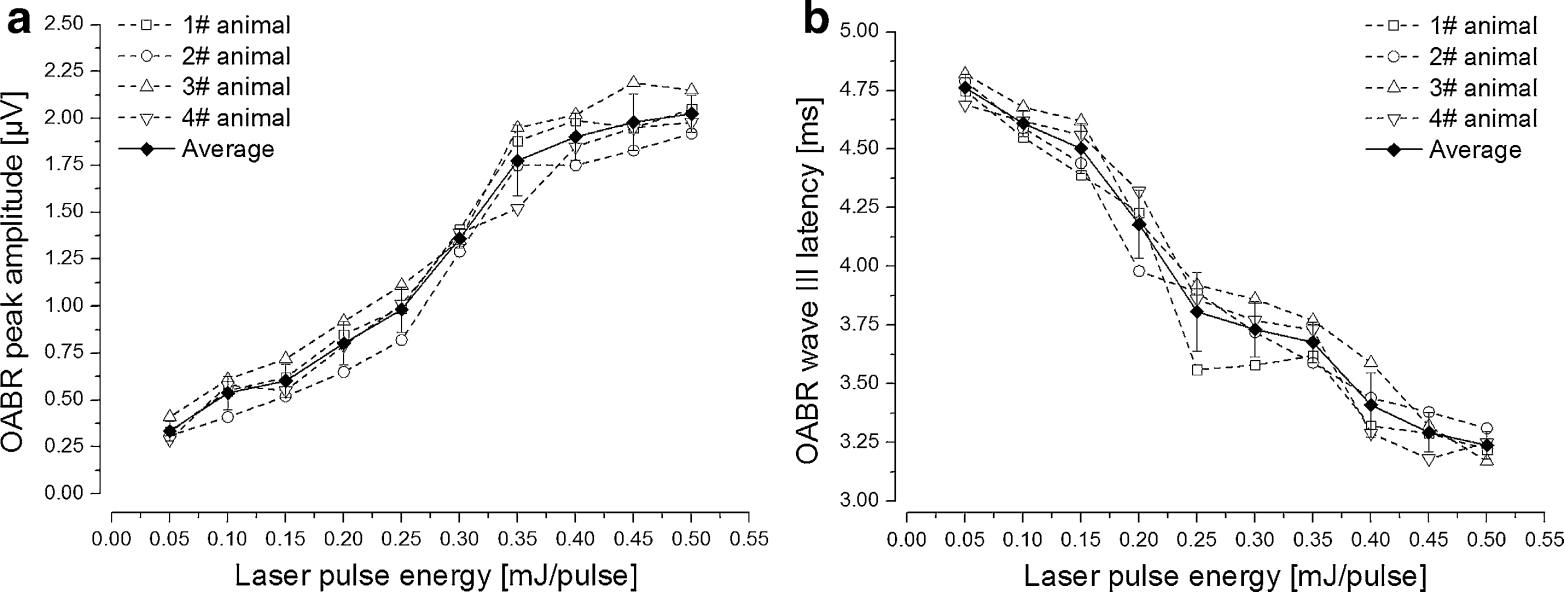
I/O curves of OABR wave III amplitude and latency as a function of laser pulse energy. The individual data measured across animals (n = 4) are shown by the different *dash lines*, and the mean is shown by the *solid diamond* with the standard error. The laser repetition rate and pulse width were kept to 11 Hz and 200 μs. And the laser pulse energy was ranged from 0.05 to 0.5 mJ/pulse by adjusting the laser output peak power from 0.25 to 2.5 W. **a** The OABR wave III peak amplitude showed a monotonic increase with the pulse energy increasing from 0.05 to 0.5 mJ/pulse. **b** The OABR wave III latency shortened along with the pulse energy increasing

After the acoustic stimulation, pulsed SWIR laser stimulation with deafened animals was conducted in two steps. With all the experimental animals, the optically induced ABR signals were recorded stably and with high repeatability, and classical Jewett waveforms have been shown distinctly. In the first step, the laser pulse repetition rate was set at 11 Hz and the pulse width was kept 200 μs. The laser pulse energy was adjusted from 0.05 to 0.5 mJ/pulse by setting the laser output peak power from 0.25 to 2.5 W. Figure 4 showed the OABR waveforms evoked with varied pulse energy levels after the deafening procedure, the waveforms were similar to the ABR evoked by acoustic stimuli, the OABR intensity increased and its wave III latency time shortened with increasing pulse energy levels. I/O curves further demonstrated this trend in Fig. 5, the OABR wave III peak amplitude increased monotonically along with the laser energy level, the mean value was 0.34 μV at 0.05 mJ/pulse energy level and it increased to 2.03 μV at 0.5 mJ/pulse laser energy, shown in Fig. 5a. And Fig. 5b showed that the OABR wave III latency time was about 4.76 ms at 0.05 mJ/pulse laser energy, while this latency shortened to 3.24 ms when the energy level increased to 0.5 mJ/pulse. Individual data across the animals were also shown in Fig. 5, and the differences were not significant (ANOVA, P > 0.05).

**Fig. 6:**
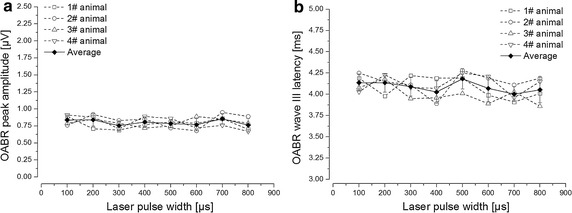
I/O curves of OABR wave III amplitude and latency as a function of laser pulse width. The individual data measured across animals (n = 4) are shown by the different *dash lines*, and the mean is shown by the *solid diamond* with the standard error. During the stimulation process, the laser pulse repetition rate was set at 11 Hz and the laser output peak power at 1 W (0.2 mJ/pulse laser pulse energy at 200 μs pulse width). The pulse width was varied from 100 to 800 μs. **a** The OABR wave III amplitude kept steady with the pulse width widened in 100–800 μs range. **b** The OABR wave III latency period basically maintained stable with the pulse width widened in 100–800 μs range

In the second optical stimulation step, we set pulse repetition rate at 11 Hz and laser output peak power at 1 W (0.2 mJ/pulse laser pulse energy at 200 μs pulse width), while varied the pulse width to investigate its effects on OABR amplitude and latency. The I/O curves showed that in the 100–800 μs pulse width range, the OABR wave III peak amplitude (0.81 ± 0.05 μV) and latency time (4.09 ± 0.09 ms) basically maintained stable, which were shown in Fig. 6a, b, respectively. The results indicated that the pulse width variation between 100 and 800 μs had no remarkable effects on the OABR parameters. The data difference between individual animals was not significant (ANOVA, P > 0.05).

## Discussion

In this study, we successfully stimulated the deafened guinea pigs auditory neurons by using the 980 nm SWIR laser with varying pulse parameters. When comparing the auditory responses, respectively from acoustic and optical stimulation, we found that the ABR wave III latency shortened along with the increase of acoustic intensity or laser pulse energy. Although there is an ABR latency shift in both acoustic and optical stimulation, the mechanisms are different. In acoustic stimulation of cochlea, the hair cells in different positions of the cochlea have various specific sensitive frequencies. When the acoustic intensity was low, only the hair cells near the sensitive frequency region could be activated. While the sound intensity increased, the activated regions of hair cells in the cochlea were broadened to the apex of cochlea, which caused some hair cells to be activated earlier than those with lower intensity. Thus, the ABR latency shortened with acoustic stimulation intensity increase. In contrast, when stimulating the auditory neurons with laser radiation, the photo-thermal effect played a role in neural activation. Laser energy was absorbed by nerve structures and transformed into heat, and the heat had to accumulate and reach the thermal gradient threshold before activating the neurons [25]. We considered that the higher laser energy might accelerate the heat accumulation process faster,then the ABR latency could be shorten. Additionally, laser radiation with higher energy intensity could enlarge the effective activation area of neurons, and the more neurons were activated, which might also lead to the latency getting shorter.

Laser stimulation performances depended on both laser parameters and tissue features [8, 18, 19, 26]. Here, the both laser parameters of pulse energy level and pulse width are mainly considered. It was observed that, along with the pulse energy increasing, OABR was stronger and the latency time shorter, which was shown in Fig. 5. In addition, the OABR wave III amplitude increasing slope became descending when the pulse energy reached a relatively high level (shown in Fig. 5). From the neuroscience viewpoint, only when the stimulation intensity exceeded the threshold, nervous impulse could be fired. The stronger stimulation intensity leaded to the stronger neural response until the saturation level was achieved, the further increasing stimulation intensity would be no longer effective to strengthen the neural response. For the SWIR laser stimulation in cochlea, we considered that it was indispensable to restrict the laser pulse energy in an appropriate range. The laser energy should be sufficient to evoke auditory neural activity, and the level should below the damage threshold [27]. Evidence has shown in previous study that laser wavelength with lower water absorption have a larger safety interval between the enough energy required for INS and the damage threshold [26], which is also an advantage of SWIR used in neural stimulation.

Furthermore, we investigated the pulse width variation effects on OABR amplitude and latency. The results showed that no obvious changes were made on OABR when varying the pulse width from 100 to 800 μs. The OABR wave III latency (4.09 ± 0.09 ms) and wave III peak amplitude (0.81 ± 0.05 μV) were almost steady (shown in Fig. 6a, b). The results indicated that the pulse width variation between 100 and 800 μs range had no remarkable influence on the OABR features. The possible mechanism may be that,after the stimulation intensity exceeded the threshold, the laser pulse peak power and the rising edge influenced the OABR, rather than the laser pulse duration. Once the laser pulse energy was deposited enough, the auditory neurons were effectively stimulated. Similar results were also presented in relevant study with longer wavelength infrared laser between 1.844 to 1.873 μm, they suggested that the laser peak power was constant across varied pulse durations ranged from 100 to 1000 μs, when evoking the same CAP intensity with 50 μV amplitude [28].

In our study, we concentrated on investigating the effects on laser pulse energy and pulse width to SWIR laser neural stimulation. The laser pulse width ranged from 100 to 800 μs, which was limited by the current laser system performance. We expect that SWIR laser with a shorter pulse width could be studied and optimized in our further experiment. Additionally, optical auditory neural stimulation in various specific areas of the cochlea could be studied in future, to investigate the small population neurons topo-reaction to laser from the basal to the apex of cochlea. And ABRs evoked by different frequencies pure-tone stimuli could serve as reference to optical stimulations. However, to support this study, additional techniques for accurately laser light delivery and spot focusing need to be developed. Moreover, appropriate methods should be made to control the laser beam divergence and optimize the spot parameter, in order to raise the optical neural stimulation efficiency and accuracy.

## Conclusions

Our study presents an auditory neural stimulation approach with the pulsed 980 nm SWIR laser. The optically induced ABRs were successfully recorded in deafened guinea pigs and the effect of pulse energy and pulse width on auditory response utilizing a SWIR laser was investigated for the first time. Results data showed that the OABR intensity increased and its latency shortened along with the pulse energy increase. And the pulse width variability in 100–800 μs range showed no discernible influence on the evoked OABR amplitude and latency period. The SWIR laser with optimized pulse parameters could be an alternative stimulus used in future optical cochlear implant.

## Abbreviations

SWIR: short-wavelength infrared
ABR: auditory brainstem response
OABR: optically evoked auditory brainstem response
INS: infrared neural stimulation
SPL: sound pressure level
ANOVA: analysis of variance
AABR: acoustically evoked auditory brainstem response
